# SARS-CoV2 mutations and impact on mortality: observational study in a sub-saharan Africa hospital

**DOI:** 10.1186/s12985-023-02014-1

**Published:** 2023-03-30

**Authors:** Jean-Robert Makulo, Roger Wumba, Madone Ndona Mandina, Placide Mbala, Adrienne Amuri Aziza, Yannick Mayamba Nlandu, Benjanmin Kabwe, Donatien Mangala, Ben Izizag Bepouka, Jerome Ossam Odio, Murielle Longokolo, Eric Mukenge, Guyguy Kamwiziku, Eddy Lusamaki Kingand, Constantin Bashengezi, Gilbert Kabanda, Benjamin Longo-Mbenza

**Affiliations:** 1 Cliniques Universitaires de Kinshasa, Université de Kinshasa, République démocratique du Congo, Kinshasa, Congo; 2 Institut National de Recherche Biomédicale (INRB), République démocratique, Kinshasa, Congo; 3 Secrétariat technique de la riposte contre la COVID-19, République démocratique du Congo, Kinshasa, Congo; 4 Centre de Recherche en phytothérapie, pharmacopée africaine et technologie pharmaceutique a (CREPPAT), Kinshasa, Congo

**Keywords:** SARS-CoV-2, Delta, Omicron, Kinshasa, DR Congo

## Abstract

**Background:**

One year after the coronavirus disease 2019 (COVID-19) pandemic, the focus of attention has shifted to the emergence and spread of severe acute respiratory syndrome coronavirus-2 (SARS-CoV-2) variants of concern (VOCs). The aim of the study was to assess the frequency of VOCs in patients followed for COVID-19 at Kinshasa university hospital (KUH) during the 3rd and 4th waves of the pandemic in Kinshasa. Hospital mortality was compared to that of the first two waves.

**Method:**

The present study included all patients in whom the diagnosis of SARS-CoV-2 infection was confirmed by the polymerase chain reaction (PCR). The laboratory team sequenced a subset of all SARS-CoV-2 positive samples with high viral loads define as Ct < 25 to ensure the chances to generate complete genome sequence. RNA extraction was performed using the Viral RNA Mini Kit (Qiagen). Depending on the platform, we used the iVar bioinformatics or artic environments to generate consensus genomes from the raw sequencing output in FASTQ format.

**Results:**

During the study period, the original strain of the virus was no longer circulating. The Delta VOC was predominant from June (92%) until November 2021 (3rd wave). The Omicron VOC, which appeared in December 2021, became largely predominant one month later (96%) corresponding the 4th wave. In-hospital mortality associated with COVID-19 fell during the 2nd wave (7% vs. 21% 1st wave), had risen during the 3rd (16%) wave before falling again during the 4th wave (7%) (p < 0.001).

**Conclusion:**

The Delta (during the 3rd wave) and Omicron VOCs (during the 4th wave) were very predominant among patients followed for Covid-19 in our hospital. Contrary to data in the general population, hospital mortality associated with severe and critical forms of COVID-19 had increased during the 3rd wave of the pandemic in Kinshasa.

## Background

The majority of viruses are characterized by mutations or deletions introduced into their genetic code. For a virus like the severe acute respiratory syndrome coronavirus-2 (SARS-CoV-2), the emergence of variants over time is therefore an expected phenomenon. Some changes do not impact the properties of the virus, however other changes can affect the virus’s properties, such as how easily it spreads, the disease severity, the performance of vaccines, the effectiveness of the drugs used, and the laboratory diagnostic tools [1, 2]. During late 2020, the emergence of variants that posed an increased risk to global public health prompted the characterization of specific Variants of Interest (VOIs) and Variants of Concern (VOCs), in order to prioritize global monitoring and research, and ultimately to inform the ongoing response to the coronavirus disease 2019 (COVID-19) pandemic [3, 4].

Among the VOCs, the SARS-CoV-2 B.1.1.7 (alpha), B.1.351 (Beta), B.1.617.2 (Delta) and B.1.1.529 (Omicron) had generated much debate during the COVID-19 pandemic around the world [3–5]. While in Europe, America and many countries of Asia, laboratories were able to identify the VOCs and VOIs during different waves of the pandemic, in sub-Saharan Africa (SSA), due to a lack of adequate resources in laboratories, these data were not available in most hospitals [6].

In the Democratic Republic of the Congo (DRC), the national laboratory had reported cases of VOCs, but analyzes were not carried out on a large scale; thus, their extent in the epidemiology of the disease is not clearly established. Taking advantage of the equipment obtained in our hospital (Viral RNA Extract Me Kit®, QiAmp Mini RNA® and a Mic RT-PCR®), the main objective of the present study was to determine the frequency of SARS-CoV-2 VOCs among patients diagnosed with COVID-19 during the 3rd and 4th waves in our hospital (from June 2021 to January 2022). The secondary objective was to compare hospital mortality associated to COVID-19 during this period versus the two previous waves of the pandemic.

## Method

The study was conducted from June 2021 to January 2022 at the Kinshasa University Hospital (KUH) and included 2254 patients in whom the diagnosis of SARS-CoV-2 infection was confirmed by the real-time polymerase chain reaction (RT-PCR). We assessed clinical and biological parameters of interest : age and sex of patient, clinical stage of COVID-19 according to the World Health Organization (WHO) classification [7], the result of the SARS-CoV-2 sequencing by specifying the type of variant and the outcome of the patient (deceased or not). During the study period, asymptomatic patients and those with mild or moderate symptoms (1411 patients) were followed on an outpatient basis, while patients with severe or critical forms of the disease were hospitalized (843 patients).

### Sample collection and laboratory diagnosis

Nasopharyngeal swab samples were placed in the universal transport medium (UTM). Detection of SARS-CoV-2 RNA was done using the GeneXpert SARS-CoV-2 test (Cepheid®).

SARS-CoV-2 positive samples with high viral loads (cycle threshold = Ct ≤ 25 for at least on of the 2 targets) were selected to maximize the chances of whole genome sequencing of the virus. The RNA extraction was performed using the Viral RNA Extract Me Kit® or QiAmp Mini RNA (Qiagen). Since the sequencing analyzes were deferred, a Mic RT-PCR® was performed to assess RNA degradation by measuring the viral load and make the sample eligible for sequencing.

Two platforms were used for sequencing:

- Illumina with Nextera DNA Flex protocol (amplicon sequencing), Miseq as sequencer and data analysis with Ivar;
- Nanopore with Artic-Gunit (amplicon sequencing) and Midnight Rapidbarcoding protocols, MinION or GridION as sequencer and analysis data on the Artic pipeline.

Consensus genomes greater than 70% were submitted in Pangolin software and those below 70% on Pango-Usher in order to identify mutations and assign the lineage. A Negative template control was included in each run to ensure the quality of sequencing. Sequences with coverage equal or greater than 80% were publicly shared and deposited to an international Global Initiative on Sharing Avian Influenza Data (GISAID) platform.

### Time period of the COVID-19 pandemic waves (Ministry of Public Health, Hygiene and Prevention, DR Congo)

The wave in a pandemic is defined as a period with the rising of the number of cases before starting to decrease in the number of cases during the period of the pandemic.

- First wave of the COVID-19 pandemic in DR Congo: week 11, year 2020 - week 37, year 2020.
-  s wave: week 44, year 2020 - week 10, year 2021.
- Third wave: week 21, year 2021-week 35, year 2021.
- Fourth wave: week 48, year 2021-week 2, year 2022.

### Statistics

All data were analyzed using SPSS for Windows version 21.00 and the OPEN EPI. We used Chi-square tests and the ANOVA test to compare, respectively, the distribution of categorical variables and the means between the groups. P < 0.05 was considered statistically significant.

### Ethical considerations

Samples were collected during the COVID-19 pandemic response activities in Kinshasa. The data were collected anonymously and confidentially. The privacy and personalities of patients have been preserved according to the Helsinki Declaration.

## Results

From the start of the pandemic until January 2022, 9930 samples had been analyzed in our laboratory for symptoms of patients suggestive of COVID-19. RT-PCR results confirmed the SARS-CoV-2 in 2254 patients (22.7%). The respective shares were: 401 (17.8%) for the 1st wave; 835 (37.1%) for the 2nd wave; 508 (22.5%) for the 3rd wave and 510 (22.6%) for the 4th wave (p < 0.001).

The Table 1 shows that the higher proportion of infected men compared to women tended to decrease during the 3rd and 4th wave (66% and 65% of male patients during the first two waves vs. 59% and 55% during the 3rd and 4th waves, p < 0.001). The proportion of hospitalized patients was higher during the 1st wave (53%) followed by the 3rd wave (49%) vs. 23% during the 2nd and 34% during the 4th wave, p < 0.001. During the 3rd and 4th wave, hospitalized patients were older than during the first two waves (respective average age of 52 ± 18 years and 58 ± 15 years vs. 61 ± 17 years and 63 ± 17 years, p < 0.001).

**Table 1:** General data on the COVID-19 pandemic in our hospital according to the waves

	1st vave	2nd wave	3rd wave	4th wave	p
Infected men, %	66	65	59	55	< 0.001
Hospitalizations, %	53	23	49	34	< 0.001
Age*, mean ± SD	52 ± 18	58 ± 15	61 ± 17	63 ± 17	< 0.001

* Hospitalized patients

**Fig. 1:**
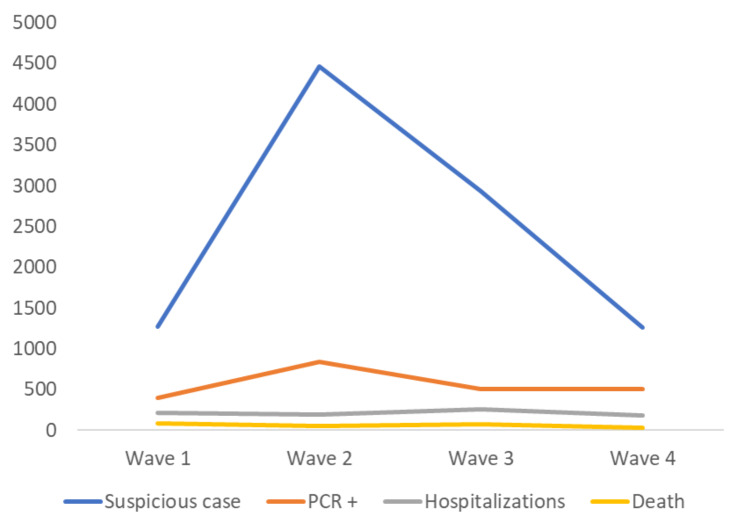
Number of cases according to the waves of COVID-19 pandemic

The number of hospitalized patients and the percentage of deaths corresponding to the total patients tested positive (hospitalized and non-hospitalized patients) were: 215 and 21% for the 1st wave, 196 and 7% for the 2nd wave, 254 and 16% for the 3rd wave and 178 and 7% for the 4th wave (p < 0.001) (Fig. 1). Among the non-hospitalized patients, no case of death had been reported.

**Fig. 2:**
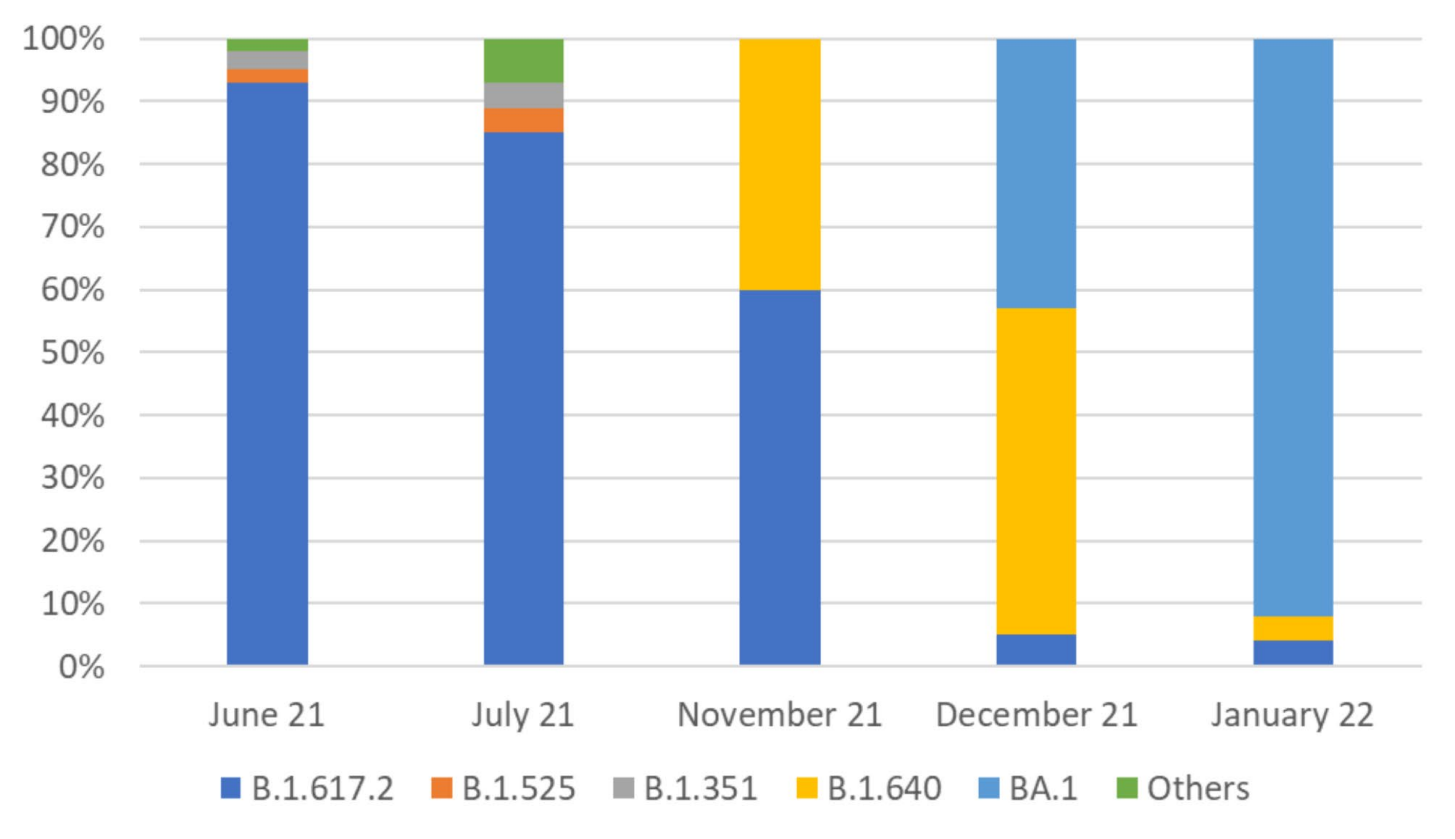
Proportion of SARS-CoV-2 variants during the 3rd and 4th waves of the COVID-19 pandemic

Our laboratory became able to search for VOCs from the 3rd and 4th wave of the pandemic in Kinshasa. Of the 269 samples received for sequencing, only 133 were produced with good coverage (greater than 50%). The other samples with low coverages did not generate sequences. The distribution of VOCs is described in Table 2; Fig. 2.

**Table 2:** Frequency of the SARS-CoV-2 variants during the 3rd and 4th waves of the pandemic

Year	Month	Sequenced samples	VOCs / VOIs						
			B.1.525	B.1.351	B.1.617.2	B.1.640	BA.1	Others	Total
2021	June	61	1	2	51	0	0	1	55
	July	29	1	1	23	0	0	2	27
	November	5	0	0	3	2	0	0	5
	December	32	0	0	1	11	9	0	21
2022	January	142	0	0	1	1	23	0	25
Total		269	2	3	79	14	32	3	133

Table 2; Fig. 2 show that the original strain from Wuhan in China was not found in the samples analyzed. The Delta VOC was mainly represented during the 3rd wave versus the Omicron during the 4th wave. The B.1.640 VOI was very present between the two periods.

## Discussion

The present study conducted in a hospital setting during the 3rd and 4 waves of the COVID-19 pandemic in Kinshasa showed a predominance of the Delta VOC, quickly supplanted by the Omicron. The original strain of the virus was practically no longer circulating. The period corresponding to the outbreak of the Delta VOC is characterized by a trend towards an increase in hospital mortality, which is nevertheless lower than the hospital mortality reported at the start of the pandemic.

The fact of finding the two VOCs respectively during the 3rd and 4th waves corroborates WHO and our national laboratory (INRB) data which had reported their presence in the DR Congo [8, 9]. What is surprising all the same are the very high proportions (Delta VOC 92% in June 2021 and Omicron VOC 96% in January 2022) as well as the absence of a transition period where we could have the cohabitation of two VOCs in equal proportions. Indeed, epidemiological reports from other countries, particularly in Europe and America, reveal that replacements of one majority VOC by another have taken place over longer periods with periods of cohabitation [10, 11]. In France, for example, despite the outbreak of the Omicron VOC, the Delta VOC had continued to circulate for a long time; in December 2021, the Delta VOC still represented half of the contaminations while the Omicron VOC was already on French territory [11]. Two factors can influence variant success: intrinsic transmissibility and immune escape [12]. The advantage of one VOC over another may depend on the immune context of the population, which varies over time (waves, vaccination campaign, immune decline) [12, 13]. Our data show that the B.1.640 VOI seems to have made the transition between the Delta VOC and the emergence of Omicron VOs. The fact that vaccination coverage was very low in the DR Congo at the time of the study may explain a more rapid expansion of each VOC.

National-level statistics report that COVID-19 lethality has declined with each wave [14]. However, our hospital data indicate that during the 3rd wave (corresponding to the emergence of the Delta VOC), mortality had increased. It was only in the 4th wave that hospital mortality fell again. Obviously, it is difficult to interpret this result. During the 3rd wave in Kinshasa, many hospitals had started taking care of COVID-19 patients. In general, these hospitals referred only more serious cases to our university hospital. We know that several studies around the world have reported greater virulence of the Delta than the Omicron VOC, however other reports have shown the opposite results [14–17]. A less virulent variant can cause several deaths in absolute value, especially if it affects a larger number of people. Apart from the fact that hospitalized patients have severe forms of the disease, the higher hospital mortality reported in this study compared to statistics for the whole country is also explained by the average age which is higher compared to that non-hospitalized patients [14].

Despite its monocentric feature and the small sample size which represent a limit, our study was able to show a great dynamic of SARS-CoV-2 with very different VOCs in the space of a few months. Thanks to genomic sequencing, the authorities have been informed of the circulation of VOCs of COVID-19 in our hospital. More resources are needed to have information for the whole country and thus contribute to better monitoring of the pandemic.

Table 1 reinforces the interest in knowing the extent of virus mutations during a pandemic. Indeed, during different waves, variabilities were observed in terms of the number of patients screened, their average age and the propensity to be hospitalized. Figure 1 also shows that hospital mortality was different depending on the waves of the pandemic although it must be admitted that mortality is influenced by several other factors.

## Conclusion

The Delta (during the 3rd wave) and Omicron VOCs (during the 4th wave) were very predominant among patients followed for Covid-19 in our hospital. Contrary to data in the general population, hospital mortality associated with severe and critical forms of COVID-19 had increased during the third wave of the pandemic in Kinshasa.

## Data Availability

The dataset supporting the conclusion of this article are available.
